# HemoMIPs—Automated analysis and result reporting pipeline for targeted sequencing data

**DOI:** 10.1371/journal.pcbi.1007956

**Published:** 2020-06-04

**Authors:** Philip Kleinert, Beth Martin, Martin Kircher

**Affiliations:** 1 Berlin Institute of Health (BIH), Berlin, Germany; 2 Charité –Universitätsmedizin Berlin, Berlin, Germany; 3 University of Washington, Department of Genome Sciences, Seattle, Washington, United States of America; Johns Hopkins University, UNITED STATES

## Abstract

Targeted sequencing of genomic regions is a cost- and time-efficient approach for screening patient cohorts. We present a fast and efficient workflow to analyze highly imbalanced, targeted next-generation sequencing data generated using molecular inversion probe (MIP) capture. Our Snakemake pipeline performs sample demultiplexing, overlap paired-end merging, alignment, MIP-arm trimming, variant calling, coverage analysis and report generation. Further, we support the analysis of probes specifically designed to capture certain structural variants and can assign sex using Y-chromosome-unique probes. In a user-friendly HTML report, we summarize all these results including covered, incomplete or missing regions, called variants and their predicted effects. We developed and tested our pipeline using the hemophilia A & B MIP design from the “My Life, Our Future” initiative. HemoMIPs is available as an open-source tool on GitHub at: https://github.com/kircherlab/hemoMIPs

## Author summary

Next generation sequencing techniques enable researchers to identify causal variants for patients in large cohorts. Targeted sequencing approaches capture genomic regions of interest to allow high throughput and cost efficient patient-specific data generation. HemoMIPs is an open-source software that analyses targeted sequencing datasets generated using molecular inversion probes (MIPs) and provides HTML reports of pathogenic and benign variants, patient sex, existence of known structural variants as well as performance statistics on the sequencing run.

This is a *PLOS Computational Biology* Software paper.

## Introduction

Patient specific variant detection is of importance for the diagnosis and treatment of various diseases. DNA capture sequencing using Molecular Inversion Probes (MIPs) is a fast and efficient method for targeted sequencing of regions of interest and has been applied in various disease cohorts [1–3]. A number of protocols exist with minor deviations from the general workflow [4]. The general approach involves designing single stranded DNA probes containing two primer sequences complementary to the region of interest as well as a linker, serving as the backbone to physically link the two primers [5]. These probes are then hybridized to the target DNA and circularized upon polymerase fill-in and nick ligation. Degradation of non-circularized molecules enriches the target DNA and sample multiplexing is enabled by using sample specific barcodes in the linker or during an amplification reaction linearizing the DNA and adding required sequencing adapters.

Hemophilia A and B are X-linked recessive disorders resulting from more than 3,000 known DNA variants in the genes encoding *coagulation factor VIII* (*F8*) and *factor IX* (*F9*), respectively. Determination of the causative genetic variant is important for the patient's reproductive planning, for use in pregnancy and neonatal management, and also to inform risks of neutralizing antibody (inhibitor) formation and bleeding severity. Therapies targeted to specific patient variants are likely to become more common in the future [6]. The "My Life, Our Future" (MLOF) project is a multisector collaboration developed to provide wide-scale access to free hemophilia genotype analysis for patients in the United States and to create a research repository of associated samples and data to support scientific discovery and treatment advances. For the MLOF initiative, a novel MIP-based genotyping approach was developed for the *F8* and *F9* genes [6] including more than 450 MIPs (see S1 Text for details).

Here, we introduce hemoMIPs, an easy and efficient pipeline to analyze MIP target capture data generated on the Illumina sequencing platform. Using an easily adapted Snakemake workflow [7], hemoMIPs performs sample demultiplexing, overlap paired-end merging, alignment using BWA, MIP-arm trimming, variant calling using GATK, coverage analysis and HTML report generation for single end and paired end sequencing datasets. While hemoMIPs was developed to analyze targeted sequencing data of the MLOF Initiative, it can be applied to a broad set of MIP sequencing data sets. Currently various tools and individual pipelines are being used in the analysis and genotyping of Molecular Inversion Probe Data. While two pipelines [2,8] are publicly inaccessible, MIPgen tools [5], bwa-MIPs [9] and MIPWrangler [10] stop after alignment and MIP-arm trimming. Therefore, hemoMIPs is the first complete analysis workflow that is open source and easy to employ via workflow management.

## Design and implementation

**Fig 1:**
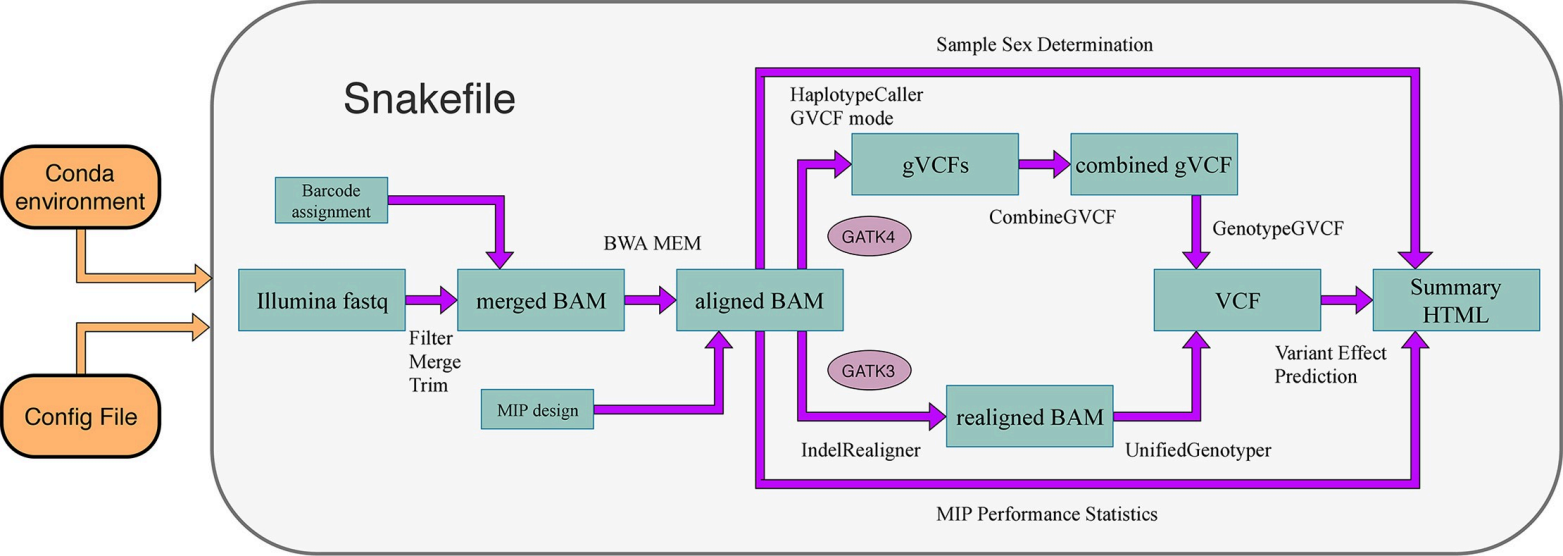
Depiction of analysis workflow of the hemoMIPs pipeline.

The hemoMIPs pipeline enables hemophilia screening of (typically) 384 patients on a single Illumina NextSeq run. Fig 1 outlines the general workflow and the following sections describe data processing and analysis in more detail. All steps are implemented in the workflow management software Snakemake [7] and rely on Conda predefined environments to manage software dependencies and easy deployment.

### Primary sequence processing

The primary inputs are raw FastQ files from the sequencing run as well as a sample-to-barcode assignment (see S1 Text). In primary processing, reads are converted to BAM format, demultiplexed (storing sample information as read group information), and overlapping paired-end reads are merged and consensus called [11].

### Alignment and MIP arm trimming

Processed reads are aligned to the reference genome (here GRCh37 build from the 1000 Genomes Project Phase II release) using Burrows-Wheeler Alignment (BWA) 0.7.5 mem [12]. As MIP arm sequence can result in incorrect variant identification (by hiding existing variation below primer sequence), MIP arm sequences are trimmed based on alignment coordinates and new BAM files are created. In this step, we are using MIP design files from MIPgen [5] by default. Alternative input formats are described in S1 Text. MIP representation statistics (text output file) are calculated from the aligned files. Further, reads aligning to the Y-chromosome-unique probes (*SRY*) are counted for each sample and reported (text output file).

In a separate alignment step, all reads are aligned to a reference sequence file describing only the structural sequence variants as mutant and reference sequences. Results are summarized over all samples with the number of reads aligning to each sequence contig in a text report.

### Coverage analysis and variant calling

Coverage differences between MIPs are handled by down sampling regions of excessive coverage. Variants are genotyped using GATK [13] UnifiedGenotyper (v3.4–46) in combination with IndelRealigner (v3.2–2). Alternatively, GATK v4.0.4.0 HaplotypeCaller is used in gVCF mode in combination with CombineGVCFs and GenotypeGVCFs.

The hemophilia datasets perform similar when run either with the GATK3 or GATK4 workflow. However, in low quality genotype calls the performance might vary and a different call set might be obtained. In a reanalysis performed on one of the hemophilia sequencing experiments, the sample specific genotype agreement is above 0.99 (36 different out of 64,308 genotype calls) between the two GATK versions, with high agreement in associated genotype qualities (S1 Fig, S1 Data). We therefore choose GATK4 as the standard setting for the workflow as this versions maintains support, is 50x faster and can be more easily upgraded.

Variant annotations of the called variants, including variant effect predictions and HGVS variant descriptions are obtained from Ensembl Variant Effect Predictor [14].

### Report generation

Different HTML reports are generated for visualization, interpretation and better access to all information collected in previous steps. There are two entry points to this information, organized as two different HTML reports–one summarizing all variant calls and MIP performance across samples and the other summarizing per-sample results in an overview table. The first report (summary.html, S2 Fig) provides a more technical sample and variant summary, per region coverage and MIP performance statistics. This report across samples can be used to assess assay performance (e.g. underperforming MIPs could be redesigned in future assays) and allows identification of suspiciously frequent variants (common variants or systematic errors).

**Fig 2:**
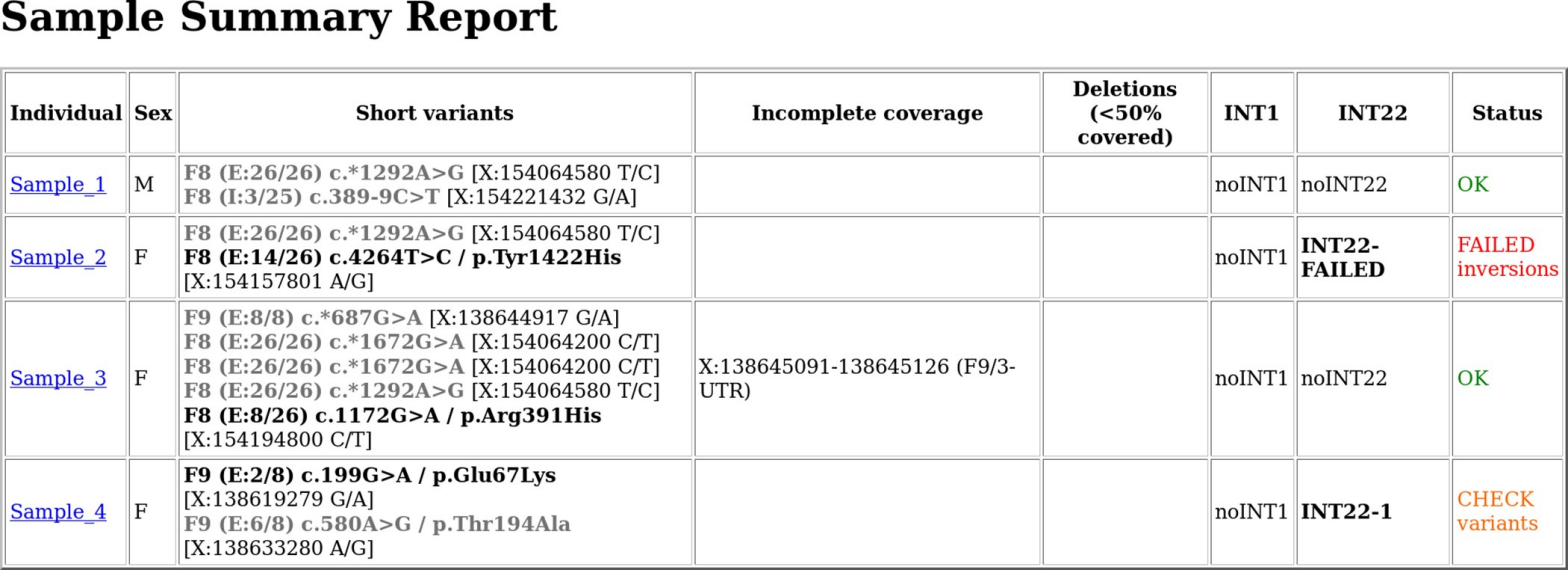
HTML reports are generated for visualization, interpretation and better access to all information collected across the individual workflow steps. Here, a section of report.html shows the obtained genotypes for the demultiplexed samples and highlights potential pathogenic variants, their location in the gene and which exon (E) or intron (I) is affected. Additionally incomplete called sites, predefined structural variants (columns INT1 and INT22 referring to inversions of F8‐intron 22 and F8‐intron 1 which are common causes of severe hemophilia A) and failed MIPs are reported. The multiplexed samples can be identified via their sampleID. This output is meant to give a general overview over the sample performances.

The second report (report.html) provides an overview of results for each sample (Fig 2), highlighting putative deleterious variants and taking previously defined common/known benign variants out of focus (gray font). Additional information is provided about potential structural variants and incompletely covered regions. This table also provides an overall sample status field with information about passing and failing samples, as well as flags indicating outlier MIP performances.

Both reports provide links to individual report pages of each sample. The individual reports (ind_SAMPLENAME.html, S3 Fig), provide quality measures like overall coverage, target region coverage, read counts underlying the inferred sample sex and MIP performance statistics (over- or underperforming MIPs in this sample), but most importantly provide detailed information on the identified variants, structural variant call results and regions without coverage (potential deletions).

### Report tables in CSV format

In additional to the HTML output files for visualization, results are also presented in computer readable CSV format files. These CSV files can be joined by either the variant or sample specific identifier columns. The following results are summarized in the respective table files:

*ind_status*.*csv* outputs the sample sex inferred from SRY counts, reports outlier MIP performance, number of genotype (GT) calls, covered sites within the MIP design regions, average coverage, heterozygous sites, incompletely covered regions, deletions as well as a textual summary in a sample quality flag (e.g. OK, Failed Inversions, Check MIPs).

*variant_calls*.*csv* and *variant_calls_benign*.*csv* contain all or just benign variants, respectively, with location, genotype, quality scores, allelic depth, coverage and status information.

*variant_annotation*.*csv* provides additional annotations to called variants based on reference and alternative allele information. These annotations include gene name, exonic location, cDNA and CDS position, HGVS Transcript and Protein information, variant rsID, and 1000G allele frequency.

*inversion_calls*.*csv* contains count results for MIPs targeting predefined structural variants.

## Results

We introduced an easy-to-use pipeline to analyze highly imbalanced, targeted next-generation sequencing data sets generated using MIP experiments. In a user-friendly HTML report, we summarize all analysis results including covered, incomplete or missing regions, called variants and their predicted effects.

Using the GATK3 version of hemoMIPs, the MLOF initiative screened 3,000 patients for hemophilia causative variants in 2017, sequencing the *F8* and *F9* genes for about 15% of the total hemophilia A and B population of the United States [6]. All *F8* and *F9* coding regions, splice sites, and upstream (450 bp for *F8* and 300 bp for *F9*) and downstream (1838 bp for *F8* and 1417 bp for *F9*) untranslated sequences were captured using 458 MIP probes, each with about 111 bp in target size. Additional eight probes were designed to capture reference or mutant sequences of large DNA inversions mediated through sequences in *F8* intron 22 or *F8* intron 1 and homologous sequences distal to the *F8* gene, resulting in gene disruptions [15]. Finally, five probes are targeting *SRY* unique sequence to detect patient sex.

In 98.4% (2,952/3,000) of patients, the likely causative variant was identified from our results and confirmed using Sanger validation [6]. Of 924 unique variants observed in this hemophilia cohort, 285 novel variants were identified. In cases of severe hemophilia, predicted gene-disrupting variants were common while missense variants dominated for mild-to-moderate disease. Novel hemophilia DNA variants were detected continuously throughout the project, indicating that additional variation likely remains undiscovered.

We have extended our pipeline to use GATK4 for variant calling and coverage analysis. Results are highly concordant between the two versions, but GATK4 calling is 50 times faster (see also S1 Text). The hemoMIPs workflow can be easily adapted to other MIP designs due to the use of Snakemake as workflow management and Conda for managing software dependencies.

## Availability and future directions

HemoMIPs is available on GitHub on https://github.com/kircherlab/hemoMIPs. Its source code is open and available for everyone to download and modify (MIT License). A manual can be found in the main repository together with example inputs and outputs to run the pipeline. All dependencies are handled by predefined conda environments available in the main repository.

As an open-source and community effort, the hemoMIPs pipeline will continue to evolve with best practice workflows (e.g. provided through GATK) as well as potential novel molecular inversion probe designs.

## Supporting information

S1 Text Additional description of the hemoMIPs pipeline and input requirements. (PDF)

S1 Data Data underlying the comparison of GATK3 and GATK4 results (S1 Fig). GATK3 vs GATK4 Genotype Quality (GQ) scores QD (Quality by Depth) scores. Both scores are on PHRED-like scale, expressing the -10*log_10_ likelihood of an incorrect call.
(XLSX)

S1 Fig Comparison of GATK3 and GATK4 results. Heatmap of GATK3 vs GATK4 Genotype Quality (GQ) scores (left) and GATK3 vs. GATK4 QD (Quality by Depth) scores (right). Both scores are on PHRED-like scale, expressing the -10*log_10_ likelihood of an incorrect call. While most variants are called with both GATK versions with high confidence (left panel, top right corner), a few variants are missed by either tool. The sample-specific genotype agreement is above 0.99 (36 different out of 64,308 genotype calls). A shifted InDel explains 6 out of 36 different genotypes. Eleven out of the remaining 30 discordant calls are seen below a total read coverage of 3 for one of the callers. Further, among the remaining discordant calls (18 out of 19 being called by GATK3), 14 are low quality calls (GQ < 30).
(TIF)

S2 Fig An example of the Summary Report. This report (summary.html) provides the user an overview of all samples present in the dataset with their inferred sex, genotypes (GT), average coverage (Ave.Cov), number of heterozygous (Hets) and overall variants and the observed variant list with direct links to the individual sample reports.
(TIF)

S3 Fig An example of an individual report. The individual report (ind_Sample_1.html) shows general quality metrics as well as functional annotations of identified variants, the coverage for each targeted region (including regions missing coverage/genotype calls), the counts for MIPs designed to capture structural variants and highlights over- or underperforming MIPs.
(TIF)
