# Supplementary material for: HemoMIPs—Automated analysis and result reporting pipeline for targeted sequencing data
Source: PLoS Comput Biol. 2020 Jun 4;16(6):e1007956. doi: 10.1371/journal.pcbi.1007956 (PMC7297380; doi:10.1371/journal.pcbi.1007956)
Supplement: S1 Text — (PDF) [file pcbi.1007956.s001.pdf]

Supporting Information

HemoMIPs – Automated analysis and result reporting pipeline for targeted sequencing data

Philip Kleinert[1,2], Beth Martin[3], Martin Kircher[1,2,3,*]

[1] Berlin Institute of Health (BIH), Berlin, Germany
[2] Charité – Universitätsmedizin Berlin, Berlin, Germany
[3] University of Washington, Department of Genome Sciences, Seattle, WA, USA

Required input files for the hemoMIPs workflow

HemoMIPs uses fastq files together with information about the MIP design and the targeted regions as input. An example dataset (`input/example_dataset`) and a detailed description of the workflow and its' configuration is available in the GitHub repository https://github.com/kircherlab/hemoMIPs.

- The required fastq input files should be created using the Illumina `bcl2fastq` tool. The pipeline can handle paired-end and single-end reads with up to two technical reads/index reads (i.e. `Undetermined_S0_L00{lane}_R1_001.fastq.gz`, `Undetermined_S0_L00{lane}_I1_001.fastq.gz`, additional for paired end read data: `Undetermined_S0_L00{lane}_R2_001.fastq.gz`, in case of a second index read: `Undetermined_S0_L00{lane}_I2_001.fastq.gz`)
- A barcode sample assignment file named `sample_index.lst`
- MIP design file as generated by https://github.com/shendurelab/MIPGEN and named `hemomips_design.txt`
- Target regions (coordinates and names) of your MIP experiment named `target_coords.bed`
- A file containing known benign variants (can be left blank) named `benignVars.txt`

Examples and further information about these files is provided below.

Barcode to sample assignment

A two or three column tab-separated file is required with the sequencing barcode information. The assigned sample name will be used throughout the processing and reporting. Multiple barcodes may assign the same sample name, causing these reads to be processed together. If a two column tab-separated file is provided, the sample barcode sequence is assumed to be in the first index read of the Illumina sequencing run (I1 fastq read file). The pipeline can also handle double index designs where sequence combinations in the I1 and I2 files identify a specific sample. Examples for the sample assignment files are provided below:

Single Index:

```
#Seq            Name
CATGCGAGA       Plate_001_01A.1
ACTGGTAGG       Plate_001_01B.2
GCTCCAACG       Plate_001_01C.3
GCGTAAGAT       Plate_001_01D.4
TGACCATCA       Plate_001_01E.5
GGATTCTCG       Plate_001_01F.6
```

Double Index:

```
#Index1         Index2          Name
GGATTCTCG       ACTGGTAGG       Plate_001_01A.1
CATGCGAGA       GCGTAAGAT       Plate_001_01B.2
TGACCATCA       TGACCATCA       Plate_001_01C.3
CATGCGAGA       GGATTCTCG       Plate_001_01D.4
```

MIP probe design information

Information about the designed MIP probes and their location in the reference genome is needed as a tab-separated text file for the tool *TrimMIParms.py*. The default input file has the following columns: index, score, chr, ext_probe_start, ext_probe_stop, ext_probe_copy, ext_probe_sequence, lig_probe_start, lig_probe_stop, lig_probe_copy, lig_probe_sequence, mip_scan_start_position, mip_scan_stop_position, scan_target_sequence, mip_sequence, feature_start_position, feature_stop_position, probe_strand, failure_flags, gene_name, mip_name. This format is obtained from MIP designs generated by MIPGEN, a tool for MIP probe design available on GitHub (https://github.com/shendurelab/MIPGEN). Alternatively, files containing at least the following named columns can be used: chr, ext_probe_start, ext_probe_stop, lig_probe_start, lig_probe_stop, probe_strand, and mip_name. It is critical, that the reported coordinates and chromosome names match the reference genome used in alignment.

Named target regions in BED format

Target regions of the MIP experiments need to be described in a BED file. These regions and names will be used in the HTML report. An example of this BED file is provided below:

```
X       154250998     154251277      F8/upstream
X       154250827     154250998      F8/5-UTR
X       154250674     154250827      F8/1
X       154227743     154227906      F8/2
...
X       154088696     154088893      F8/25
X       154065871     154066037      F8/26
X       154064063     154065871      F8/3-UTR
X       154064033     154064063      F8/downstream
```

Benign variants

Known benign variants can be described in `benignVars.txt`. If no such variants are available, an empty file with this name needs to be provided. If variants are provided in this file, these will be printed in gray font in the HTML report (see manuscript Fig 2 and S3 Fig) and separated in the CSV output files. An example of the variant format is provided below.

```
X_138633280_A/G
X_154065069_T/G
X_138644836_G/A
X_138645058_GT/-
X_138645060_-/GT
X_138645149_T/C
```

Configuring the MIP project

Different aspects of the project (e.g. sequencing run information, reference sequences, local paths, benign variants) can be defined using a central configuration file (`config.yml`) for the snakemake workflow. An example is available at https://github.com/kircherlab/hemoMIPs/blob/master/example_config.yml

Providing information about the sequencing setup

Specify whether the sequencing was performed using a single-index or double-index design, single or paired-end reads and whether specific inversion MIPs are provided.

Providing reference and tool path information

Different references and annotations need to be specified, such as reference genome, BWA indexed reference, inversion reference, and VEP installation path and cache version.

Providing run information

As multiple Illumina lanes can be analyzed simultaneously, the respective run folder names in the local "input" folder and the number of lanes can be set in the "datasets" section of the configuration file. Further, the version of GATK (gatk3, gatk4, or both) can be selected and the variant reporting limited to certain transcripts.

Other supported MIP read layouts

While hemoMIPs was developed to analyze targeted sequencing data of the MLOF Initiative (PE 120bp, 8bp single index), it can be applied to a broad set of MIP sequencing data sets. Other layouts such as single end sequencing and up to two technical reads can be used. From the technical reads either the first (single index) or both identify the sample (double index). If no double indexing is specified, but a second technical read is specified, its sequence is propagated with the other read information. Thereby, UMI information can be maintained throughout the processing and later evaluated. If UMI sequences are actually read as part of paired end or single end reads, these might be moved to the second technical read. If double indexing is also used, the two double index sequences might be combined into one virtual read, freeing the second technical read for UMIs. For a detailed description of how to preprocess such alternative read formats before applying our workflow, please see the section on alternative read layouts in our manual (https://github.com/kircherlab/hemoMIPs/).
