# Supplementary figures and images for: HemoMIPs—Automated analysis and result reporting pipeline for targeted sequencing data
Source: PLoS Comput Biol. 2020 Jun 4;16(6):e1007956. doi: 10.1371/journal.pcbi.1007956 (PMC7297380; doi:10.1371/journal.pcbi.1007956)

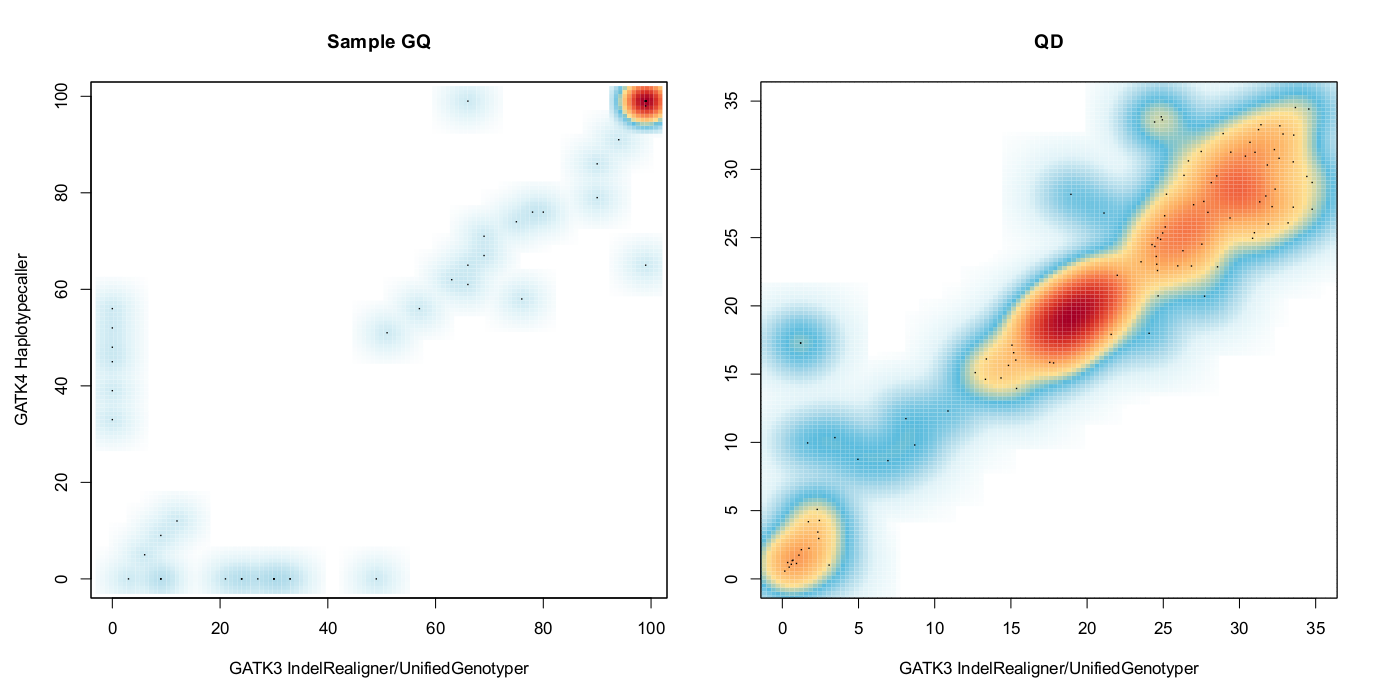

Supplement: S1 Fig — Heatmap of GATK3 vs GATK4 Genotype Quality (GQ) scores (left) and GATK3 vs. GATK4 QD (Quality by Depth) scores (right). Both scores are on PHRED-like scale, expressing the -10*log10 likelihood of an incorrect call. While most variants are called with both GATK versions with high confidence (left panel, top right corner), a few variants are missed by either tool. The sample-specific genotype agreement is above 0.99 (36 different out of 64,308 genotype calls). A shifted InDel explains 6 out of 36 different genotypes. Eleven out of the remaining 30 discordant calls are seen below a total read coverage of 3 for one of the callers. Further, among the remaining discordant calls (18 out of 19 being called by GATK3), 14 are low quality calls (GQ < 30). (TIF) [file pcbi.1007956.s003.tif]

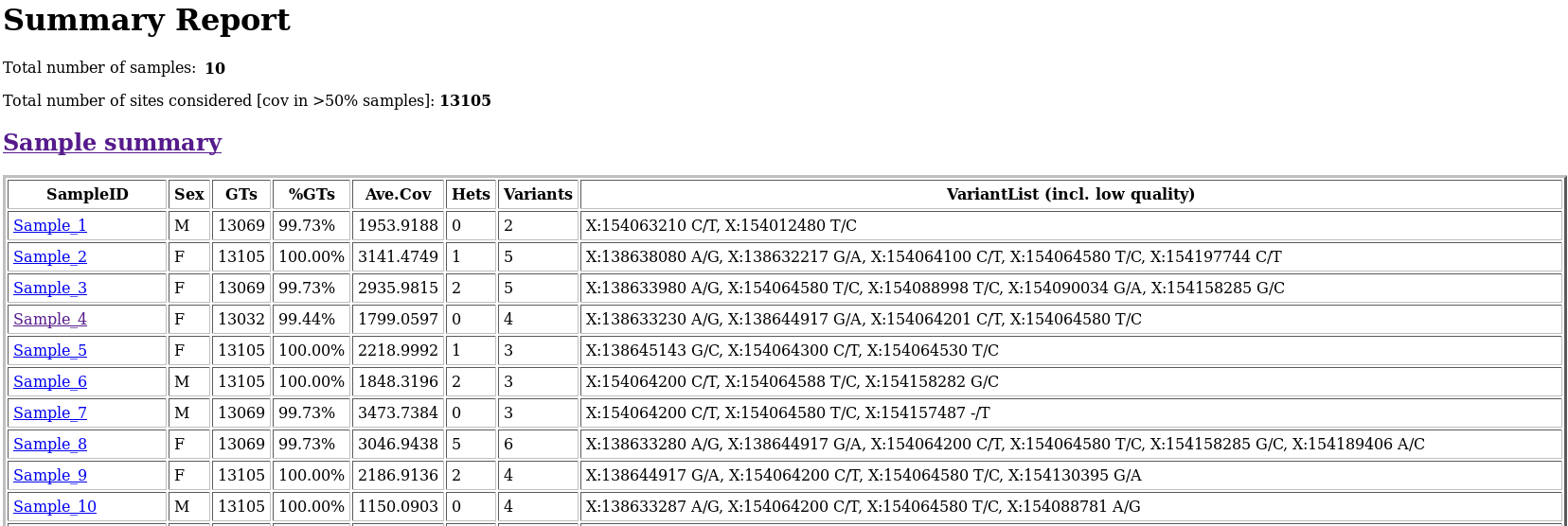

Supplement: S2 Fig — This report (summary.html) provides the user an overview of all samples present in the dataset with their inferred sex, genotypes (GT), average coverage (Ave.Cov), number of heterozygous (Hets) and overall variants and the observed variant list with direct links to the individual sample reports. (TIF) [file pcbi.1007956.s004.tif]

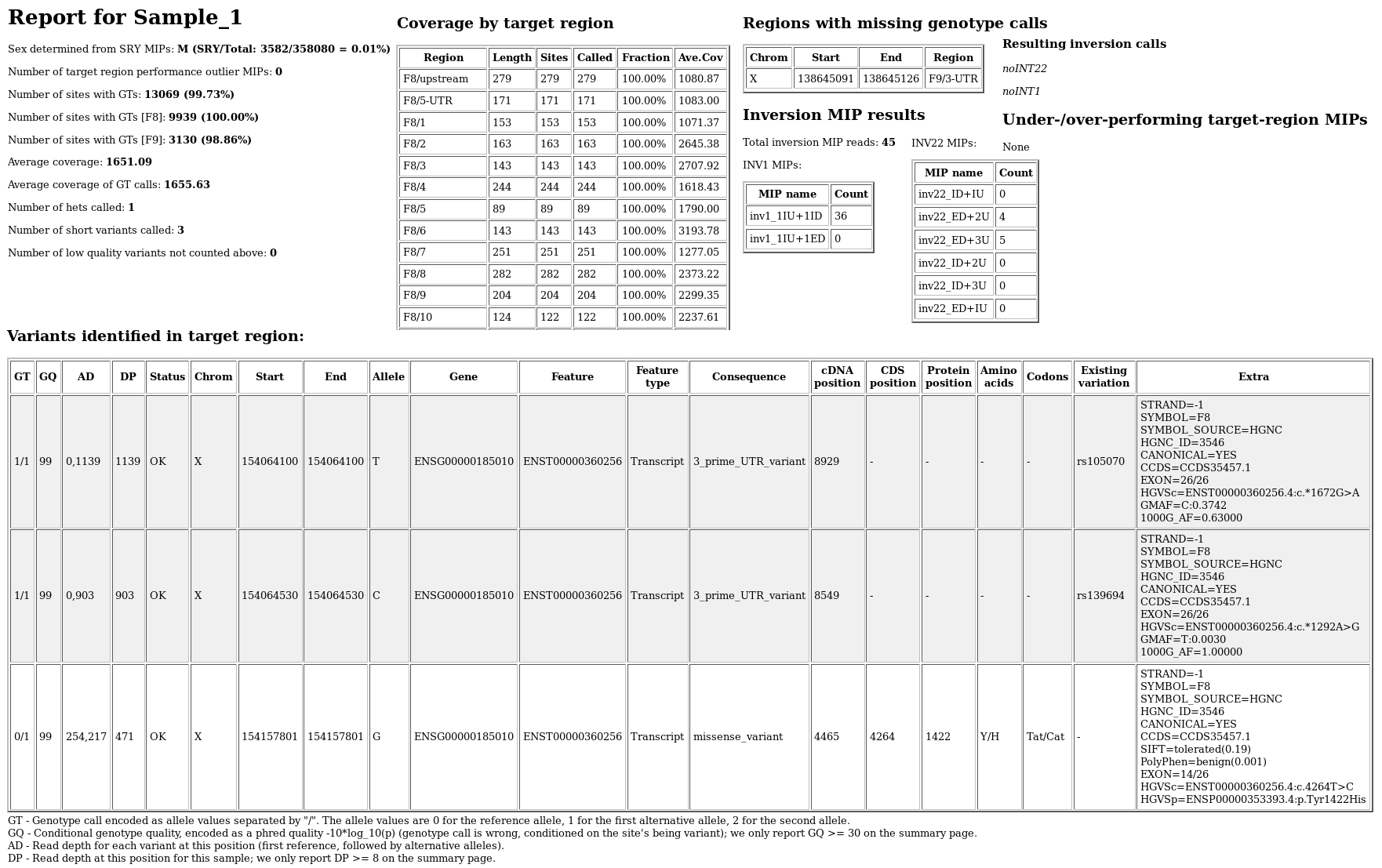

Supplement: S3 Fig — The individual report (ind_Sample_1.html) shows general quality metrics as well as functional annotations of identified variants, the coverage for each targeted region (including regions missing coverage/genotype calls), the counts for MIPs designed to capture structural variants and highlights over- or underperforming MIPs. (TIF) [file pcbi.1007956.s005.tif]
